# Timing and Frequency Synchronization Using CAZAC Sequences for OFDM Systems

**DOI:** 10.3390/s23063168

**Published:** 2023-03-16

**Authors:** Gang Peng, Rui Li, Yushu He, Zhiren Han

**Affiliations:** 1 College of Information and Communication Engineering, Harbin Engineering University, Harbin 150001, China; 2 Wuhan Maritime Communication Research Institute, Wuhan 430200, China

**Keywords:** orthogonal frequency division multiplexing (OFDM), timing synchronization, correlation operation, Zadoff–Chu (ZC) sequence, frequency offset estimation, fast Fourier transform (FFT)

## Abstract

Since orthogonal frequency division multiplexing (OFDM) systems are very susceptible to symbol timing offset (STO) and carrier frequency offset (CFO), which cause inter-symbol interference (ISI) and inter-carrier interference (ICI), accurate STO and CFO estimations are very important. In this study, first, a new preamble structure based on the Zadoff–Chu (ZC) sequences was designed. On this basis, we proposed a new timing synchronization algorithm, called the continuous correlation peak detection (CCPD) algorithm, and its improved algorithm: the accumulated correlation peak detection (ACPD) algorithm. Next, the correlation peaks that were obtained during the timing synchronization were used for the frequency offset estimation. For this, the quadratic interpolation algorithm was adopted as the frequency offset estimation algorithm, which was better than the fast Fourier transform (FFT) algorithm. The simulation results showed that when the correct timing probability reached 100%, under the parameters of *m* = 8 and *N* = 512, the performance of the CCPD algorithm was 4 dB higher than that of Du’s algorithm, and that of the ACPD algorithm was 7 dB. Under the same parameters, the quadratic interpolation algorithm also had a great performance improvement in both small and large frequency offsets, when compared with the FFT algorithm.

## 1. Introduction

Orthogonal frequency division multiplexing (OFDM), as a high-performance physical layer technology of wireless communication, has been widely used in wireless communication systems for its advantages of high spectrum utilization, high power utilization, and strong resistance to multipath delay expansion and frequency selective fading [1]. Specifically, IEEE 802.11 uses OFDM in wireless local area network (LAN) applications, and 802.16 uses OFDM in wireless network applications [2]. However, OFDM systems are very susceptible to symbol timing offset (STO) and carrier frequency offset (CFO) [3]. STO can result in inter-symbol interference (ISI) between two adjacent OFDM symbols, which leads to phase rotation. Meanwhile, CFO can cause inter-carrier interference (ICI), which destroys the orthogonality between the subcarriers [4]. These problems seriously degrade the bit error rate (BER) performance of communication systems. Therefore, accurate STO and CFO estimations are very important in OFDM systems.

In practical OFDM systems, the estimation of STO and CFO, also called the timing synchronization and the frequency synchronization, are achieved by sending preambles at the transmitter and by processing the received signals at the receiver [5]. The preambles usually adopt one or more pseudo-noise (PN) sequences; the most commonly used one is the m-sequence. At the transmitter, the preamble waveforms are modulated and transmitted separately from the data waveforms as training signals [6]. At the receiver, the methods for timing synchronization can be classified into two categories. One is based on the autocorrelation of the received signals. The training sequences are repeated sequences, and the receiver performs a delayed autocorrelation on the received signal sequences and then determines the timing synchronization position using the autocorrelation peak. The other one is based on the cross-correlation of the received signals. The receiver performs a sliding cross-correlation between the received signal sequences and the known training sequences, so that the sharp correlation peak can be obtained at the timing synchronization point and so that it is easy to determine the accurate synchronization position [7].

Based on the literature search of the related works, we found that using CAZAC sequences as a preamble for synchronization in an OFDM system is more effective than using PN sequences. Furthermore, the correlation value is proportional to the length of the preamble; therefore, a longer preamble can achieve higher synchronization estimation accuracy. On the other hand, the frequency offset has a greater impact on a longer preamble. In order to reduce this effect, segmenting the preamble is a common and effective method. This is the source of the research idea of this paper. The innovation points and main contributions of this paper are summarized as follows:

1. A new preamble structure based on ZC sequences was designed;
2. On this basis, a new timing synchronization algorithm, called the continuous correlation peak detection (CCPD) algorithm, was proposed;
3. In addition, an improved algorithm of the CCPD algorithm, called the accumulated correlation peak detection (ACPD) algorithm, was proposed;
4. Next, the correlation peaks that were obtained during the timing synchronization were used for the frequency offset estimation;
5. The frequency offset estimation algorithm adopted the quadratic interpolation algorithm, which was improved from the FFT algorithm.

The remainder of this paper is organized as follows: In Section 2, the related works in recent years are introduced. In Section 3, some necessary background is provided, including the OFDM signal model, the CAZAC sequences, and the FFT frequency estimation algorithm. In Section 4, first, a new preamble structure is presented. Based on this foundation, a new timing synchronization algorithm and its improved algorithm are proposed. Next, the frequency offset is estimated by using the correlation peaks extracted by the synchronization algorithm. In Section 5, the performance of the proposed algorithms is simulated and compared with previous algorithms. In Section 6, the main conclusions are summarized, and the paper’s limitations and future works are given.

## 2. Related Works

In recent decades, related signal processing techniques have been extensively researched, and many pioneering results have been obtained. In 1997, Schmidl and Cox [8] proposed a method of timing and frequency synchronization by using a training sequence of two symbols; however, the timing metric function curve of the estimator had a platform, which made the timing synchronization estimation ambiguous. In 2000, Minn et al. [9] proposed a new structure of the training sequences, involving a sharp synchronous autocorrelation peak. This eliminated the plateau effect of Schmidl’s method. However, Minn’s method still suffered from the problem of time ambiguity due to the existence of many sidelobe peaks on both sides of the main synchronous correlation peak of the timing metric function curve. In 2003, Park et al. [10] proposed a new preamble structure and correlation method, which obtained an impulse-like shaped timing metric function curve and, thus, further improved the timing accuracy. However, under the conditions of a low signal-to-noise ratio (SNR), Park’s method still had two large sidelobes, which resulted in an unsatisfactory timing performance and a large estimation error. In 2008, Yi et al. [11] designed a new training symbol and proposed a novel time and frequency synchronization scheme, in which the timing metric function curve had a distinct peak and which also eliminated the sidelobes. In 2016, Nasir et al. [12] surveyed the literature from 2010 to 2014 and presented a comprehensive literature review and classification of the latest research progress in the timing and frequency synchronization techniques that had been proposed for different communication systems. They focused on the system model conceptions for synchronization, the synchronization challenges, and the most advanced synchronization methods and their limitations. In 2018, Abdzadeh-Ziabari et al. [13] proposed a novel preamble-assisted approach for the joint estimation of timing, carrier frequency offset, and channels in OFDM systems that operate under high mobility conditions. In addition, an efficient algorithm was proposed to reduce the computational complexity of joint estimation. In 2019, Hu et al. [14] considered a consensus problem of high-order multiagent systems with antagonistic interactions and communication noises. In the same year, Salih Abdelgader et al. [15] proposed a new timing synchronization scheme for OFDM communication systems, which exploited the phase shifts caused by timing errors and the power differences among the subcarriers. In the same year, Du et al. [16] proposed a new timing estimation algorithm by designing a new structure of training symbols. The simulation results showed that the algorithm could obtain accurate STO estimations due to its timing metric, which had a sharp and clear peak. In 2020, Yang et al. [17] presented a robust timing synchronization method based on a fast timing search window and a dual threshold decision. The fast timing search window effectively reduced the probability of false alarms, and the dual threshold judgment reduced the probability of acquisition loss. Furthermore, simulations were performed to verify the effectiveness of the proposed strategy. In 2021, Guo et al. [18] proposed a novel 5G-based centralized switch fault current limiter framework as well as a method to allocate such flexible fault current limiters optimally. In the same year, Jung et al. [19] proposed a simple method for estimating STO and fractional CFO in cyclic prefix (CP) OFDM-based systems. A CP-based correlation function was proposed in the pre-fast Fourier transform (FFT) synchronization to avoid the effects of ISI, which are caused by multipath fading channels, and the effects of ICI, which are caused by the performance differences in local oscillators between the transmitter and the receiver. In 2022, Li et al. [20] introduced a control problem with unique finite/fixed-time stability considerations, namely time-synchronized stability, and accordingly, presented sufficient conditions for it. In the same year, Yuan et al. [21] studied the CFO synchronization technology in OFDM, analyzed CFO in the time and frequency domains, and simulated the performance of CFO synchronization technology based on OFDM.

Compared with PN sequences, constant amplitude zero autocorrelation (CAZAC) sequences had better autocorrelation and cross-correlation properties [22]. Therefore, many synchronization algorithms that are based on CAZAC sequences were proposed in succession. In 2005, Ren et al. [23] proposed a new timing and frequency offset estimation method. This was based on a new constant envelope preamble that was generated by the discrete Fourier transform (DFT) of a CAZAC sequence. This method almost eliminated the sidelobes and significantly improved the accuracy of the timing offset estimation. Ren’s method performed well under additive white Gaussian noise (AWGN) channels; however, its performance degraded significantly under multipath fading channels. New synchronization algorithms, which were based on CAZAC sequences, were proposed by Fang and Shao in [24,25]. In both, they used different weighting factors from those of Ren, which improved the synchronization performance under multipath fading channels, effectively utilized the properties of CAZAC sequences, and achieved stable and accurate combined timing and frequency synchronization for OFDM systems. In 2015, Gul et al. [26] proposed a timing and frequency synchronization algorithm using the Zadoff–Chu (ZC) sequence (one type of CAZAC sequence). This had joint signal detection, timing synchronization, and CFO estimation, which simplified the complete synchronization structure and improved the synchronization performance. In 2017, Colombo et al. [27] designed a preamble, which was based on Golay sequences and CAZAC sequences, and they proposed a low-complexity timing and frequency synchronization algorithm for OFDM systems. When compared with the existing alternative methods, this novel scheme achieved competitive performance and significantly reduced computational complexity. In 2018, Chung et al. [28] developed new CAZAC and near CAZAC sequences to enable robust fine timing and frequency synchronization, which endowed OFDM preamble waveforms with a compact spectrum and a low peak-to-mean envelope power ratio (PMEPR). In the same year, Lin et al. [29] proposed a new near-CAZAC sequence to construct spectrally compact OFDM preamble waveforms. The new waveforms had robust timing and frequency synchronization and very low PMEPR, compared to waveforms that had been built using traditional CAZAC sequences. In 2019, Yang et al. [30] proposed a new synchronization algorithm, which was based on scrambling sequences, to improve the synchronization performance of OFDM systems in the presence of interference. This redesigned the training sequence with CAZAC sequences and additional scrambling operations. Compared with the traditional algorithm based on scrambling sequences, the simulations showed that the proposed synchronization algorithm could maintain a sharper correlation peak in an interference environment and could achieve accurate STO and CFO estimations. In 2020, Fan et al. [31] proposed a time–frequency synchronization scheme that was based on a new synchronization preamble, using CAZAC sequences. This effectively improved the stability and accuracy of OFDM system synchronization. In 2021, Palanisamy et al. [32] built a compact band-pass filter with improved band-stop and band-pass characteristics for wireless applications with four internal conductive poles in a single resonating cavity, which added novel quad-resonating modes to the realization of the band-pass filter. In 2022, Tomar et al. [33] presented the challenges/problems for supporting multipath transmission with possible solutions and then reviewed recent research efforts related to the concurrent multipath transmission protocol and the multipath transmission control protocol. In the same year, Chen et al. [34] considered a finite-time velocity-free rendezvous control method for multiple autonomous underwater vehicle systems with intermittent undirected communication. In the same year, Berggren et al. [35] proposed a waveform in which the orthogonal basis functions were constructed from cyclically shifted versions of the zero autocorrelation (ZAC) sequences. They deduced the relevant properties and found that this waveform was also suitable as a synchronization signal, since the modulation symbols could be chosen to obtain the ideal periodic autocorrelation function (PACF) and low PMEPR.

Based on the above literature search, we found that the previous synchronization algorithms usually detect only one correlation peak. This can easily cause false synchronization and large timing errors in a low SNR. In this paper, the proposed synchronization algorithms require the detection of multiple correlation peaks, and the distance between adjacent correlation peaks should be equal. These are the main improvements of the proposed algorithms over previous ones.

## 3. Preliminaries

In this section, some necessary preliminary knowledge is introduced, including the OFDM signal model, the CAZAC sequences, and the FFT frequency estimation algorithm.

### 3.1. OFDM Signal Model

**Figure 1:**
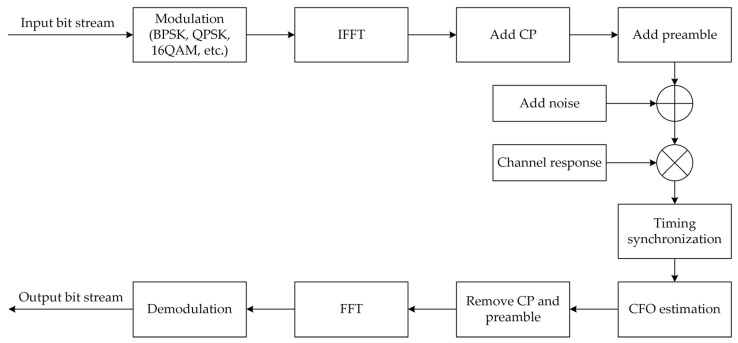
General model of an OFDM system.

Consider a general model of an OFDM system, which is shown in Figure 1. The *n*th sample of the transmitted baseband signals in the time domain can be described as

(1) $$s\left(n\right)=\frac{1}{\sqrt{N}}\sum_{k=0}^{N_{u}-1}c_{k}e^{j2\pi kn/N},\;\left(-N_{cp}\leq n\leq N-1\right),$$

where *N* is the number of inverse fast Fourier transform (IFFT) points, and it also denotes the total number of subcarriers, $N_{u}$ is the number of effective subcarriers, $j=\sqrt{-1}$, and $C_{k}$ is the modulated data sample on the *k*th subcarrier. To prevent ISI from destroying the orthogonality between the subcarriers, we usually put a copy of the last $N_{cp}$ length samples of the OFDM symbol at the head of the time-domain symbol as a CP.

At the receiver, the *n*th sample of the received signal is given by

(2) $$r\left(n\right)=\sum_{l=0}^{L-1}h_{l}s\left(n-\tau_{l}-d\right)\cdot e^{j2\pi fn/N}+w\left(n\right),$$

where *L* is the number of the channel paths, $\tau_{l}$ is the delay of the *l*th channel path, *d* is the integer-valued timing offset, *f* is the normalized frequency offset, and w(n) is the sample of AWGN.

### 3.2. CAZAC Sequences

CAZAC sequences have many excellent characteristics, including constant amplitude, ideal periodic autocorrelation and good cross-correlation properties, and low PMEPR. In addition, they are still CAZAC sequences after the forward and inverse Fourier transform. Therefore, CAZAC sequences are usually used as training sequences for symbol synchronization.

The most commonly used CAZAC sequences include ZC sequences [36], Frank sequences [37], Golomb polyphase sequences [38], and generalized chirp-like (GCL) sequences [39]. Among these, ZC sequences are the most widely used.

The ZC sequences can be described as

(3) $$a_{k}\left(N,u,q\right)=\{\begin{array}{c} \exp\left(\frac{j2\pi u}{N}\left(\frac{k^{2}}{2}+qk\right)\right),\;for\;N\;even\\ \exp\left(\frac{j2\pi u}{N}\left(\frac{k\left(k+1\right)}{2}+qk\right)\right),\;for\;N\;odd \end{array},$$

where *N* is the length of the ZC sequences, k=0,1,⋯,N−1, *q* is an arbitrary integer, and *u* is a positive integer coprime to *N*.

Let $a_{k}$ be a ZC sequence of length $N=sm^{2}$ as defined in Equation (3), where *s* and *m* are any positive integers, and then the GCL sequences are defined as

(4) $$s_{k}=a_{k}b_{\left(k\right)\;\mathrm{mod}\;m},\;k=0,1,\cdots ,N-1,$$

where $b_{i},\;i=0,1,\cdots ,m-1$ is a sequence containing *m* complex numbers, whose absolute value is equal to one, and (*k*) mod *m* means that index *k* is reduced modulo *m*. Here, it is easy to see that ZC sequences are exactly the GCL sequences when m=1 and $b_{i}=1,\;i=0,1,\cdots ,m-1$.

CAZAC sequences have better autocorrelation and cross-correlation properties than traditional PN sequences. The autocorrelation function (ACF) of the CAZAC sequences can be given as

(5) $$\sum_{k=0}^{N-1}c\left(k\right)\cdot c^{*}\left(k+\tau \right)=\{\begin{array}{c} 0,\;\tau \neq 0\\ N,\;\tau =0 \end{array},$$

where c(k) is a CAZAC sequence with a period of *N*, and * means the conjugate operation.

### 3.3. FFT Frequency Estimation Algorithm

The received signal with noise after the timing synchronization can be described as

(6) $$r\left(n\right)=Ae^{j2\pi fnT_{s}}+w\left(n\right),$$

where $T_{s}$ is the sampling period, *f* is the frequency offset, and w(n) is the sample of AWGN.

The FFT frequency estimation algorithm is used to estimate the carrier frequency *f* by using the FFT operation on the received signal r(n). We perform the $N_{F}$-point FFT operation on the received signal r(n) to obtain Y(n)=FFT(r(n)) and then perform an $N_{F}/2$-point spectrum shift on Y(n) to obtain $Y_{shift}\left(n\right)=FFTshift\left[Y\left(n\right)\right]$. Then we can obtain the corresponding frequency point of the maximum value of $Y_{shift}\left(n\right)$ as

(7) $$k_{\max}=\left\{k|\max\left[Y_{shift}\left(k\right)\right],0\leq k\leq N_{F}-1\right\},$$

which is the relative offset position of the maximum due to the frequency offset.

Then the absolute offset position of the maximum due to frequency offset is

(8) $$k_{\mathrm{CFO}}=k_{\max}-N_{F}/2$$

and the estimated value of the frequency offset can be obtained as

(9) $${\hat{f}}_{\mathrm{FFT}}=\frac{k_{\mathrm{CFO}}}{N_{F}}\cdot f_{s},$$

where $f_{s}$ is the sampling frequency and $f_{s}=1/T_{s}$.

## 4. Materials and Methods

In this section, a new preamble structure is designed first. On this basis, we propose a new timing synchronization algorithm and its improved algorithm. Next, the correlation peaks extracted by the synchronization algorithm are used to estimate the frequency offset.

### 4.1. Synchronization Preamble Design

In a practical communication system, a preamble is usually added before each frame of data to be sent at the transmitter. At the receiver, the received signal is correlated with the local sequences, and the exact position of the frame header is determined by detecting the correlation peak. PN sequences are often used as a preamble. CAZAC sequences have better autocorrelation and cross-correlation properties than traditional PN sequences. As can be seen from Equation (5), the correlation peak value is proportional to the length of the CAZAC sequences; therefore, it is easier to detect the frame header with a longer preamble. On the other hand, the existence of CFO will affect the performance of correlation peak detection. The longer the preamble, the greater the influence of CFO; therefore, it is better for the preamble to be shorter.

**Figure 2:**
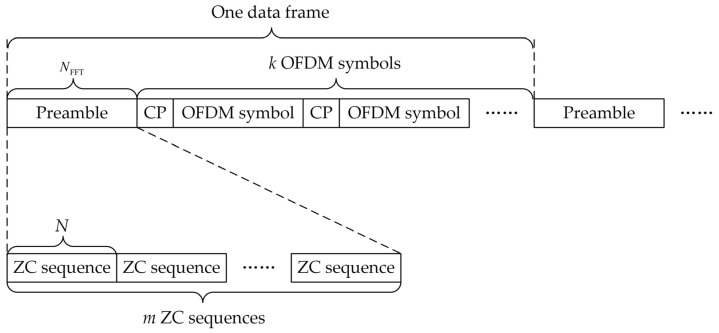
The structure of the new preamble based on the ZC sequences.

In order to reduce the impact of CFO, while still guaranteeing the performance of the correlation peak detection, a novel preamble based on the ZC sequences is proposed. The structure of the new preamble is shown in Figure 2.

It can be seen from Figure 2 that a data frame is composed of a preamble and *k* OFDM symbols (including CP). The preamble consists of *m* identical ZC sequences of length *N*, and *m* and *N* satisfy $N=N_{\mathrm{FFT}}/m$, where $N_{\mathrm{FFT}}$ is the length of the OFDM symbols.

### 4.2. Timing Synchronization Method

Based on the new synchronization preamble, a novel timing synchronization method is proposed, which utilizes the ideal correlation properties of the ZC sequences.

**Figure 3:**
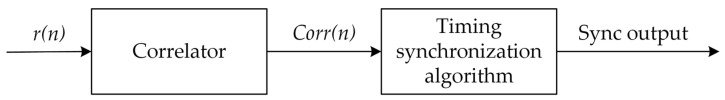
The proposed timing synchronization method.

The proposed method is composed of two stages. In the first stage, the ZC sequence that makes up the preamble is used as the coefficients of the correlator. The received signal r(n) is then input into the correlator to perform a correlation operation with the ZC sequence. Finally, the correlation value signal Corr(n) is output. In the second stage, the correlation value signal Corr(n) is used for synchronization detection. The schematic of the proposed method is shown in Figure 3.

Through the correlation value signal that is output by the correlator, we propose a new timing synchronization algorithm as follows.

#### 4.2.1. Continuous Correlation Peak Detection Algorithm

The correlator output signal Corr(n) is the result of the correlation between the received signal r(n) and the ZC sequence, which can be described as

(10) $$Corr(n)=\sum_{k=0}^{N-1}r(n-N+k)\cdot a^{*}(k),$$

where the received signal r(n) is shown in Equation (2) and the ZC sequence a(n) is described by Equation (3).

**Figure 4:**
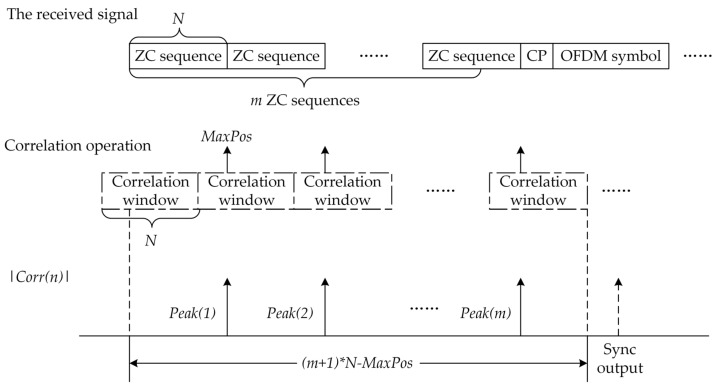
The amplitude variation of the correlation value Corr(n).

The synchronization position is obtained by detecting the peak of Corr(n). The amplitude variation of Corr(n) is shown in Figure 4, where |Corr(n)| is the absolute value of Corr(n).

The operation period of the correlator is the length *N* of the ZC sequences in the preamble, and one operation period is called a correlation window. As the correlator continuously outputs the signal, the continuous correlation peaks of |Corr(n)| can be detected, and their number is *m*, which is the number of ZC sequences in the preamble. At the same time, the intervals between these correlation peaks are identical and equal to the ZC sequence length *N*. The position of these correlation peaks from the starting point of the corresponding correlation window is *MaxPos*.

Based on the regular positions of these correlation peaks, we propose a new timing synchronization algorithm, called the continuous correlation peak detection (CCPD) algorithm. The process of the CCPD algorithm is as follows:

- Perform correlation operation on the received signal r(n) and the ZC sequence to obtain the correlation value Corr(n);
- Set a correlation window with a period of *N*, where the parameter *Counter* (1≤Counter≤N) indicates the relative position of each point in the correlation window, and record the relative position *MaxPos* of each correlation peak in the corresponding correlation window and its peak value (denoted as *PeakValue*);
- If the *MaxPos* of the current correlation window is the same as that of the one before (denoted as *MaxPosBefore*), then add one to the parameter *MaxPosTimes*; otherwise, set it to zero;
- If *MaxPosTimes* is greater than m−1, the received signal has been synchronized successfully. Next, calculate the frame header position of the received signal according to the *MaxPos*. It is easy to calculate the distance between the synchronization decision point and the frame header as (m+1)∗N−MaxPos.

**Figure 5:**
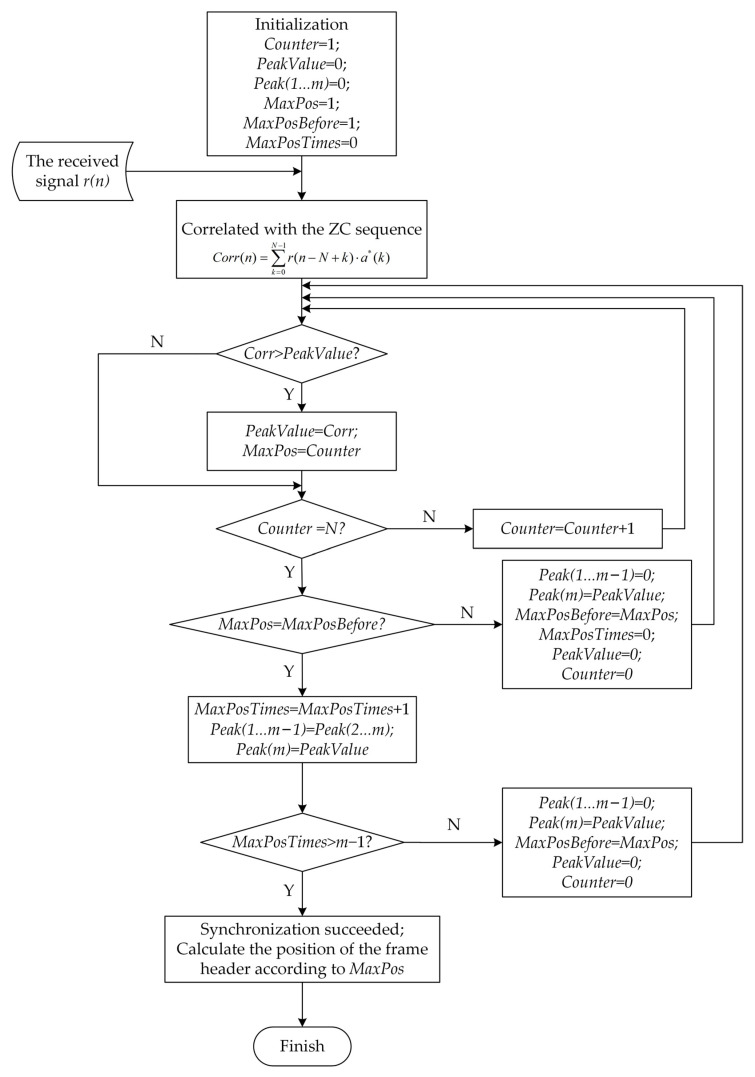
The flowchart of the continuous correlation peak detection (CCPD) algorithm.

In order to understand the process of the CCPD algorithm more intuitively, the algorithm flowchart is drawn as shown in Figure 5.

#### 4.2.2. Accumulated Correlation Peak Detection Algorithm

We improve the CCPD algorithm above and propose a new timing synchronization algorithm that performs better. This is called the accumulated correlation peak detection (ACPD) algorithm.

Similar to the CCPD algorithm, the correlation value Corr(n) is obtained first. Next, *m* correlation values are accumulated with an interval of *N* to obtain CorrSum(n), which can be described as

(11) $$CorrSum\left(n\right)=\sum_{i=0}^{m-1}\left|Corr\left(n-i*N\right)\right|.$$

**Figure 6:**
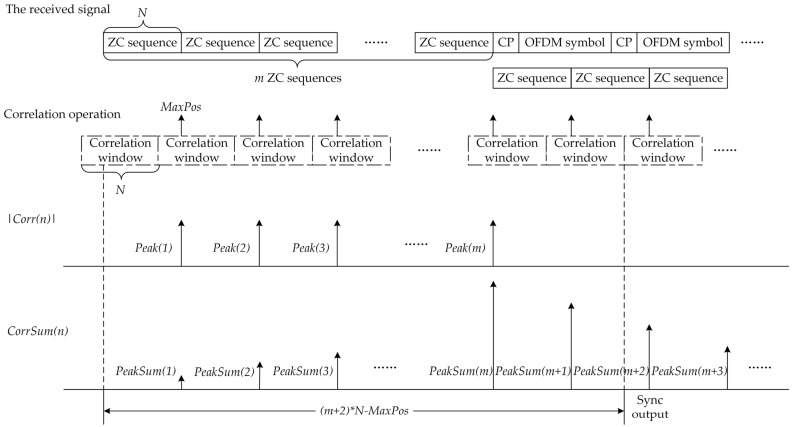
The amplitude variation of CorrSum(n).

The synchronization position is obtained by detecting the peak of CorrSum(n). The amplitude variation of CorrSum(n) is shown in Figure 6.

As can be seen from Figure 6, the peak value of CorrSum(n) first increases and then decreases. If the position of the maximum correlation peak can be found, the position of the frame header can be calculated accordingly. The process of the ACPD algorithm is as follows:

- Perform correlation operation on the received signal r(n) and the ZC sequence to obtain the correlation value Corr(n);
- Accumulate *m* correlation values with an interval of *N* to obtain CorrSum(n);
- Set a correlation window with a period of *N*, where the parameter *Counter* (1≤Counter≤N) indicates the relative position of each point in the correlation window, and record the relative position *MaxPos* of each correlation peak in the corresponding correlation window and its peak value (denoted as *PeakValue*), as well as that of the one before (denoted as *PeakValueBefore*);
- If the *MaxPos* of the current correlation window is the same as that of the one before (denoted as *MaxPosBefore*), then add one to the parameter *MaxPosTimes*; otherwise, set it to zero;
- If *MaxPosTimes* is greater than *Threshold*, and *PeakValue* is smaller than *PeakValueBefore*, the received signal has been synchronized successfully. The parameter *Threshold* is the threshold value of the number of detected correlation peaks, which varies with the number *m* of ZC sequences. Next, calculate the frame header position of the received signal according to *MaxPos*. It is easy to calculate the distance between the synchronization decision point and the frame header as (m+2)∗N−MaxPos.

**Figure 7:**
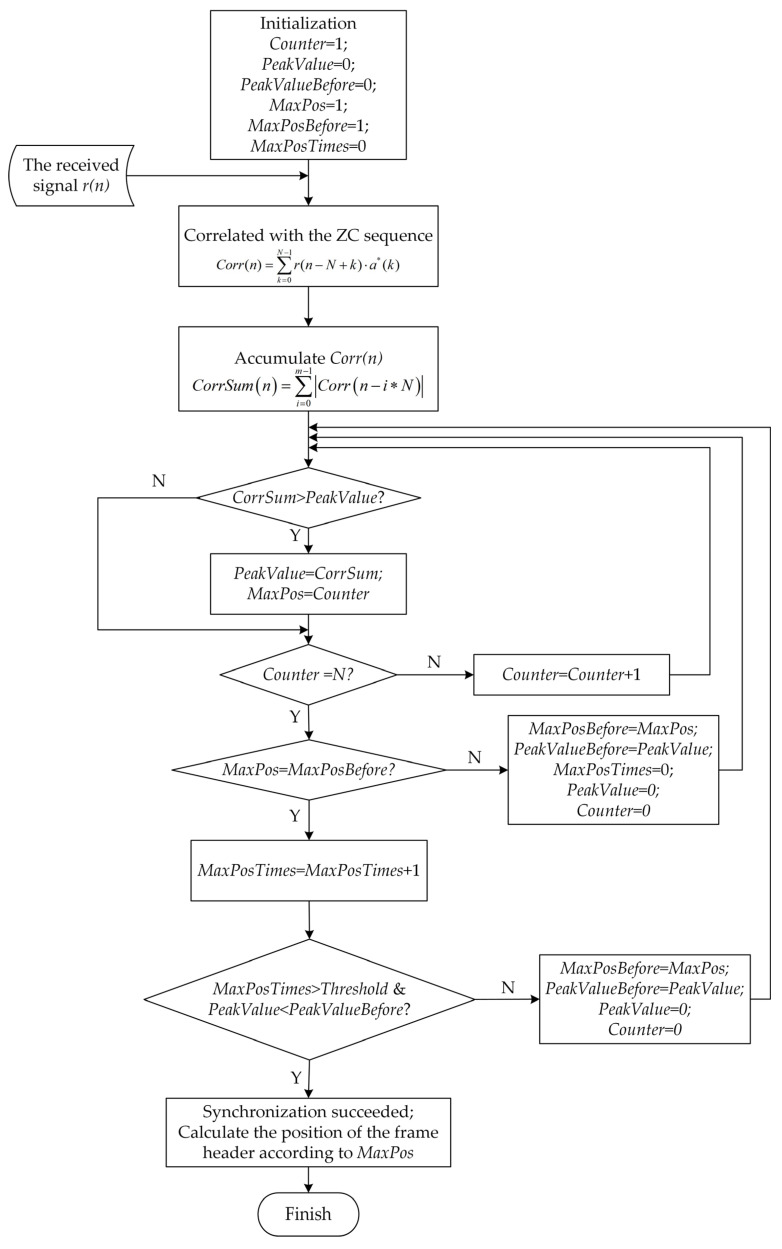
The flowchart of the accumulated correlation peak detection (ACPD) algorithm.

The flowchart of the ACPD algorithm is shown in Figure 7.

### 4.3. Frequency Offset Estimation Method

According to the above timing synchronization algorithms, the *m* correlation peak value of Corr(n) can be obtained as follows: Peak(i),i=1,2,⋯,m. Next, $N_{F}-m$ zeros are filled behind these *m* peaks, and then the $N_{F}$-point FFT operation is performed on the resulting sequence, and the FFT results are used for frequency estimation. The obtained sequence can be represented as $Corr_{\mathrm{FFT}}(k)=\mathrm{FFT}\left(\left[Peak\left(1\ldots m\right);zeros\left(1\ldots N_{F}-m\right)\right]\right),0\leq k\leq N_{F}-1$.

Since the accuracy of the FFT frequency estimation algorithm is not high enough, it can only be used for coarse estimation [40]; however, we use the quadratic interpolation frequency estimation algorithm to solve this problem. The quadratic interpolation algorithm is based on the coarse estimation of the FFT algorithm, and it uses the two values on both sides of the maximum value to perform fine estimation, according to the interpolation formula.

According to the FFT algorithm, we can obtain the maximum value $Corr_{\mathrm{FFT}}\left(k_{\mathrm{FFT}}\right)$ of the spectrum energy and its corresponding frequency point $k_{\mathrm{FFT}}$. At the same time, we can obtain two values $Corr_{\mathrm{FFT}}\left(k_{\mathrm{FFT}}-1\right)$ and $Corr_{\mathrm{FFT}}\left(k_{\mathrm{FFT}}+1\right)$ on both sides of the maximum value $Corr_{\mathrm{FFT}}\left(k_{\mathrm{FFT}}\right)$, and their corresponding frequency points $k_{\mathrm{FFT}}-1$ and $k_{\mathrm{FFT}}+1$. From these three points, we can construct a quadratic curve $z=ak^{2}+bk+c$ and then substitute the three points into *z* to obtain the coefficients *a*, *b*, and *c*. By calculating the maximum value $Corr_{\mathrm{FFT}}\left(k_{\max}\right)$ of curve *z* and its corresponding frequency point $k_{\max}$, we can obtain a more accurate frequency estimation value ${\hat{f}}_{\mathrm{int}}$. We can easily obtain the formula for $k_{\max}$ as

(12) $$k_{\max}=k_{\mathrm{FFT}}+\frac{3Corr_{\mathrm{FFT}}\left(k_{\mathrm{FFT}}-1\right)-4Corr_{\mathrm{FFT}}\left(k_{\mathrm{FFT}}\right)+Corr_{\mathrm{FFT}}\left(k_{\mathrm{FFT}}+1\right)}{2Corr_{\mathrm{FFT}}\left(k_{\mathrm{FFT}}-1\right)-4Corr_{\mathrm{FFT}}\left(k_{\mathrm{FFT}}\right)+2Corr_{\mathrm{FFT}}\left(k_{\mathrm{FFT}}+1\right)}-1,$$

which is the relative offset position of the maximum due to the frequency offset.

As in the FFT algorithm, the absolute offset position of the maximum due to frequency offset is

(13) $$k_{\mathrm{CFO}}=k_{\max}-N_{F}/2,$$

and then the estimated value of the frequency offset can be obtained as

(14) $${\hat{f}}_{\mathrm{int}}=\frac{k_{\mathrm{CFO}}}{N\cdot N_{F}}\cdot f_{s},$$

where *N* is the length of the ZC sequences, $N_{F}$ is the FFT operation points, and $f_{s}$ is the sampling frequency.

**Figure 8:**
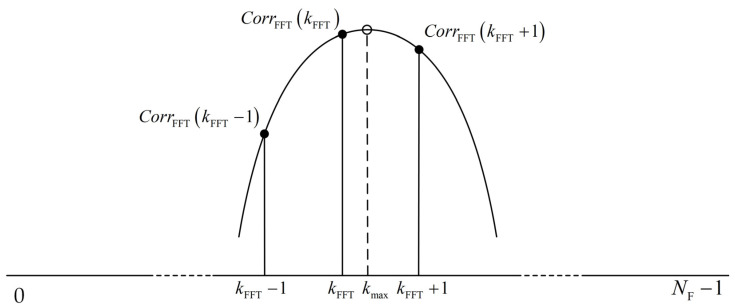
The schematic diagram of the quadratic interpolation algorithm.

The schematic diagram of the quadratic interpolation algorithm is shown in Figure 8.

Since $k_{\max}\in \left[0,N_{F}\right)$, we can obtain $-N_{F}/2\leq k_{\mathrm{CFO}}<N_{F}/2$. By combining this with Equation (14), we can obtain the frequency offset estimation range as ${\hat{f}}_{\mathrm{int}}\in \left[-\frac{f_{s}}{2N},\frac{f_{s}}{2N}\right)$, where *N* is the length of the ZC sequences in the preamble.

## 5. Results and Discussion

In this section, the performance of the proposed CCPD and ACPD timing synchronization algorithms is simulated and compared to previous algorithms.

The simulation system model adopted the OFDM system shown in Figure 1. The main system parameters are summarized in Table 1.

**Figure 9:**
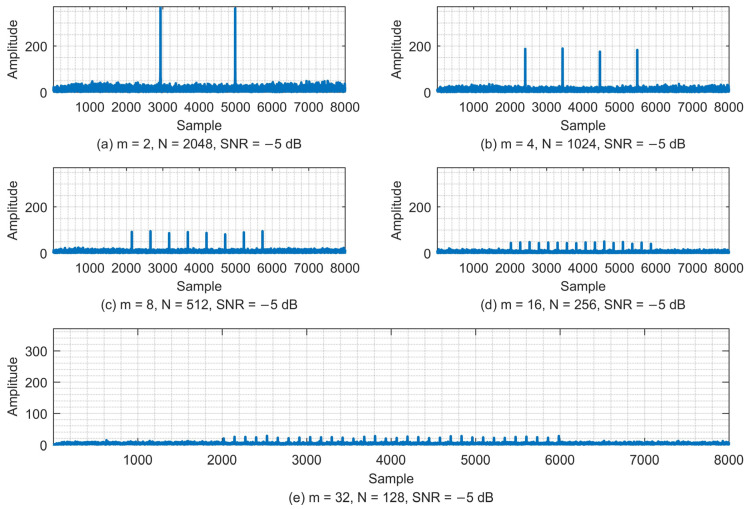
(**a**) The amplitude of the correlation value Corr(n) when *m* = 2, *N* = 2048, and SNR = −5 dB. (**b**) The amplitude of the correlation value Corr(n) when *m* = 4, *N* = 1024, and SNR = −5 dB. (**c**) The amplitude of the correlation value Corr(n) when *m* = 8, *N* = 512, and SNR = −5 dB. (**d**) The amplitude of the correlation value Corr(n) when *m* = 16, *N* = 256, and SNR = −5 dB. (**e**) The amplitude of the correlation value Corr(n) when *m* = 32, *N* = 128, and SNR = −5 dB.

The amplitude variation of the correlation value Corr(n) is simulated under different values of *m* and *N*, as shown in Figure 9. The number *m* values of ZC sequences are 2, 4, 8, 16, and 32, and the corresponding length *N* values of the ZC sequences are 2048, 1024, 521, 256, and 128, respectively, so that they satisfy $m*N=N_{\mathrm{FFT}}=4096$.

In the case that $N_{\mathrm{FFT}}$ remains constant, as the number *m* of ZC sequences increases, the length *N* of the ZC sequences decreases accordingly. This, in turn, leads to a decrease in the correlation value Corr(n). This trend of change is clearly shown in Figure 9.

### 5.1. Simulation of the CCPD Algorithm

The CCPD algorithm has two important parameters: the number *m* and length *N* of the ZC sequences included in the preamble. In the following, the impact of different *m* and *N* on the performance of the CCPD algorithm is simulated.

**Figure 10:**
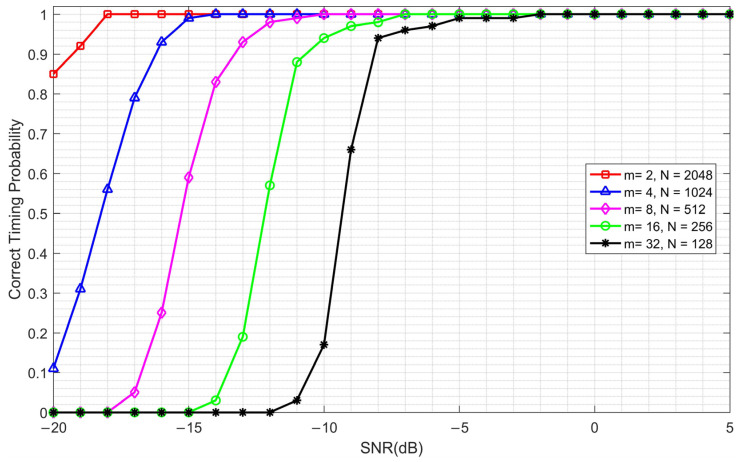
The correct timing probability of the CCPD algorithm with different *m* and *N* under a fixed CFO of 4 kHz.

A fixed CFO of 4 kHz is added to the simulation system, and the correct timing probability of the CCPD algorithm with different *m* and *N* is shown in Figure 10.

From Figure 10, we can see that, on the whole, the synchronization accuracy of the CCPD algorithm increases as the SNR increases. In the case of a low SNR, the synchronization accuracy decreases as *N* decreases. This is because as *N* decreases, the amplitude of the correlation peaks also decreases. At a low SNR, the correlation peaks are submerged in the noise and, thus, are more difficult to examine, which can be seen in Figure 9. However, when the SNR increases above −2 dB, there is no difference in the synchronization accuracy with different *m* and *N*, and all synchronization accuracy values reach 100%. This is because as the SNR increases, the correlation peaks become more prominent. Therefore, in the case of a high SNR, the synchronization accuracy is not affected by the variation in *m* and *N*.

From the above discussion, we seem to be able to draw the following conclusion: the larger *N* is (and, correspondingly, the smaller *m* is), the better the performance of the CCPD algorithm will be. However, this conclusion is not comprehensive, because the performance of the frequency offset estimation has not been considered.

In Section 4.3, we concluded that the range of the frequency offset estimation of the quadratic interpolation algorithm was $\left[-\frac{f_{s}}{2N},\frac{f_{s}}{2N}\right)$, which was inversely proportional to the length *N* of the ZC sequences. Since the sampling frequency is 100 MHz, we can calculate that the frequency offset estimation range is ±24.414 KHz when *N* = 2048, ±48.828 KHz when *N* = 1024, ±97.656 KHz when *N* = 512, ±195.312 KHz when *N* = 256, and ±390.625 KHz when *N* = 128.

**Figure 11:**
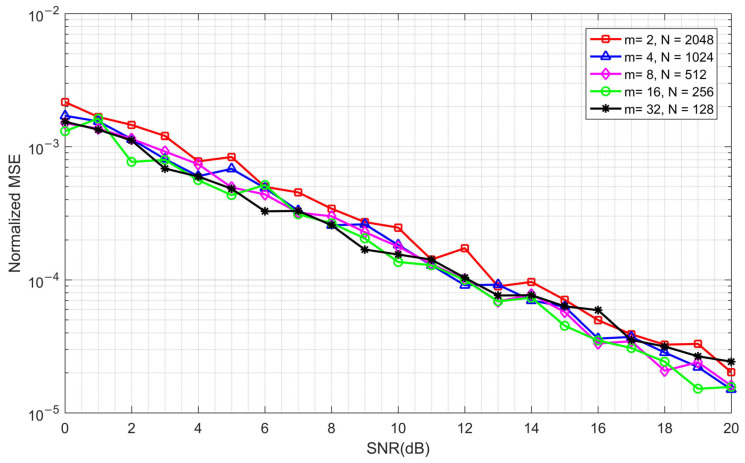
The normalized mean square error (MSE) with different *m* and *N* under a fixed CFO of 4 kHz.

In the following, the normalized mean square error (MSE) of the frequency offset estimation is simulated for different *m* and *N*. First, a small CFO of 4 KHz is added to the simulation system, which is far smaller than the upper bound of the minimum estimation range (mentioned above). The simulation results are shown in Figure 11.

It can be seen from Figure 11 that when the CFO is much smaller than the estimation range, the accuracy values of the frequency offset estimations under different *m* and *N* are almost the same.

**Figure 12:**
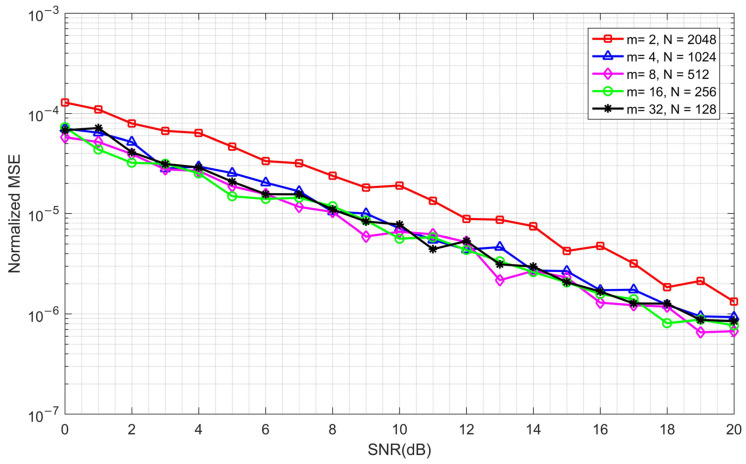
The normalized MSE with different *m* and *N* under a fixed CFO of 20 kHz.

Next, a CFO of 20 KHz is added to the simulation system, which is close to the upper bound of the estimation range for *m* = 2 and *N* = 2048. The simulation results are shown in Figure 12.

It can be seen from Figure 12 that the accuracy of the frequency offset estimation when *m* = 2 and *N* = 2048 is worse than that in other cases. This is because the CFO is close to the upper bound of the estimation range, which causes the estimation performance to approach the limit, thus causing performance degradation.

By combining Figure 10, Figure 11 and Figure 12, it can be seen that the timing synchronization accuracy and the frequency offset estimation range are contradictory. Therefore, if we want to obtain the best performance, we must make a compromise. In a practical communication system, the optimal parameters need to be determined according to the possible frequency offset range. Under the simulation conditions presented in Table 1, the frequency offset range is ±97.656 KHz when *m* = 8 and *N* = 512, which can meet the requirements of most communication scenarios. Therefore, we use these parameters in subsequent simulations.

### 5.2. Comparison of the CCPD Algorithm and the ACPD Algorithm

**Figure 13:**
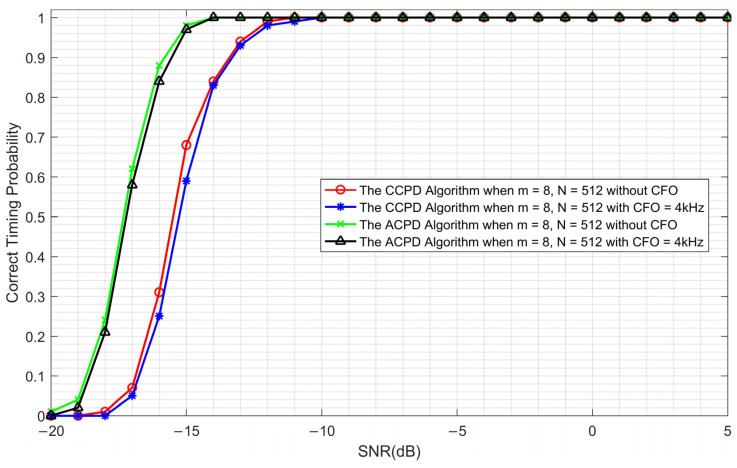
Correct timing probability comparison of the CCPD algorithm and the ACPD algorithm.

The performance of the ACPD algorithm is simulated and compared to the CCPD algorithm. The number *m* of the ZC sequences is 8, the length *N* is 512, and the CFOs are zero and 4 kHz, respectively. The simulation results are shown in Figure 13.

It can be seen from Figure 13 that the existence of CFO weakened the performance of both the CCPD algorithm and the ACPD algorithm; however, its impact is negligible. Furthermore, under the same correct timing probability, the ACPD algorithm has a performance improvement of 2 dB to 3 dB, when compared with the CCPD algorithm.

### 5.3. Comparison of the Proposed Algorithms with Previous Algorithms

**Figure 14:**
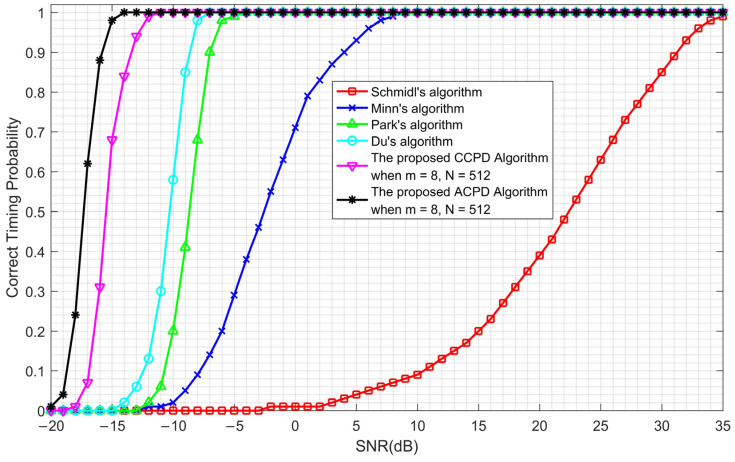
Correct timing probability comparison of the proposed algorithms with previous algorithms.

The performance of our two proposed algorithms is simulated and compared with previous algorithms, including Schmidl’s algorithm [8], Minn’s algorithm [9], Park’s algorithm [10], and Du’s algorithm [16]. The preambles of the previous algorithms and those of the proposed algorithms are summarized in Table 2. The simulation results are shown in Figure 14.

As can be seen in Figure 14, the correct timing probability of Du’s algorithm reaches 100% when the SNR is greater than −7 dB, while the CCPD algorithm reaches 100% when the SNR is greater than −11 dB. This is a performance improvement of 4 dB. Furthermore, the ACPD algorithm reaches 100% at −14 dB, which is 7 dB better than Du’s algorithm. Therefore, both the CCPD and ACPD algorithms showed a great improvement in performance when compared with the previous algorithms.

### 5.4. Comparison of the FFT Algorithm and the Quadratic Interpolation Algorithm

**Figure 15:**
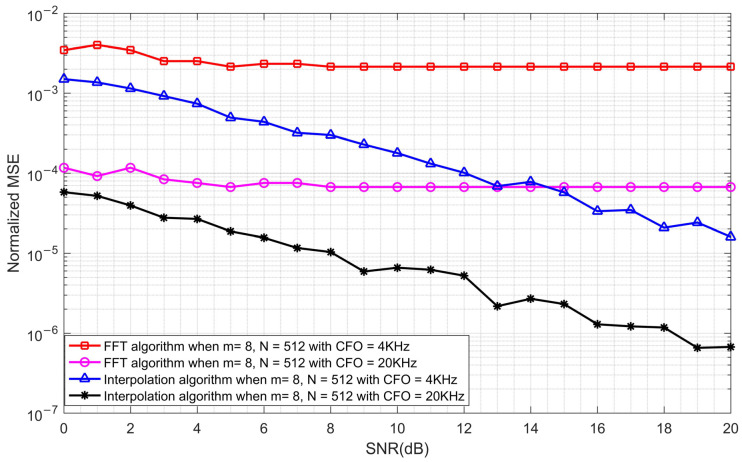
The normalized MSE comparison of the FFT algorithm and the quadratic interpolation algorithm when *m* = 8 and *N* = 512 with CFOs of 4 KHz and 20 KHz.

The CFO estimation performance of the FFT algorithm and the quadratic interpolation algorithm is simulated and compared. The number *m* of ZC sequences is 8, the length *N* is 512, and CFOs are 4 kHz and 20 kHz, respectively. The simulation results are shown in Figure 15.

As can be seen from Figure 15, when the frequency offset is far from the boundary of the estimation range, the estimation performance is better at a large frequency offset. Furthermore, the quadratic interpolation algorithm showed a great improvement in performance when compared with the FFT algorithm, in both small and large frequency offsets.

## 6. Conclusions

In this paper, first, a new preamble structure based on ZC sequences for OFDM systems was presented. On this basis, a novel timing synchronization algorithm—the CCPD algorithm—and its improved algorithm—the ACPD algorithm—were proposed. Next, the correlation peaks that were obtained during the timing synchronization were used for the frequency offset estimation. The frequency offset estimation algorithm adopted the quadratic interpolation algorithm, which was improved from the FFT algorithm.

We simulated the performance of the proposed algorithms at different values of number *m* and length *N* of ZC sequences, and then we compared their performance with previous algorithms. The simulation results showed that the timing synchronization accuracy was contradictory to the frequency offset estimation range in the choice of parameters *m* and *N*; therefore, we need to make a compromise. When the correct timing probability reached 100%, under the parameters of *m* = 8 and *N* = 512, the performance of the CCPD algorithm was 4 dB higher than that of Du’s algorithm, and the performance of the ACPD algorithm was 7 dB higher than that of Du’s algorithm. Under the same parameters, the quadratic interpolation algorithm also showed a great improvement in performance in both small and large frequency offsets, when compared with the FFT algorithm.

This paper did not consider the effect of phase offset on the performance of the proposed algorithm, because we have not yet proposed a more efficient phase offset estimation algorithm. Furthermore, the frequency offset estimation algorithm proposed in this paper had the limitation of estimation range. These are the problems that need to be solved in the future.

## Figures and Tables

**Table 1:** The parameters of the simulation system.

Parameter	Value
signal bandwidth	40 MHz
sampling frequency	100 MHz
modulation method	QPSK
length of OFDM symbols	4096
CP length	290
simulation times	100

**Table 2:** The preambles of different algorithms.

Algorithm	Preamble
Schmidl’s algorithm [8]	$\left[A_{N/2},A_{N/2}\right]$
Minn’s algorithm [9]	$\left[A_{N/4},A_{N/4},-A_{N/4},-A_{N/4}\right]$
Park’s algorithm [10]	$\left[A_{N/4},B_{N/4},A^{*}{}_{N/4},B^{*}{}_{N/4}\right]$
Du’s algorithm [16]	$\left[A_{N/4},-B^{*}{}_{N/4},-B^{*}{}_{N/4},A_{N/4}\right]$
The CCPD algorithm	$\left[A_{N/m},A_{N/m},\cdots ,A_{N/m}\right]$
The ACPD algorithm	$\left[A_{N/m},A_{N/m},\cdots ,A_{N/m}\right]$

## Data Availability

The data used to support this study will be available from the corresponding author on reasonable request.

## Abbreviations

The following abbreviations are used in this manuscript:

ACF	Autocorrelation Function
ACPD	Accumulated Correlation Peak Detection
AWGN	Additive White Gaussian Noise
BER	Bit Error Rate
CAZAC	Constant Amplitude Zero Autocorrelation
CCPD	Continuous Correlation Peak Detection
CFO	Carrier Frequency Offset
CP	Cyclic Prefix
DFT	Discrete Fourier Transform
FFT	Fast Fourier Transform
GCL	Generalized Chirp-Like
ICI	Inter-Carrier Interference
IFFT	Inverse Fast Fourier Transform
ISI	Inter-Symbol Interference
LAN	Local Area Network
MSE	Mean Square Error
OFDM	Orthogonal Frequency Division Multiplexing
PACF	Periodic Autocorrelation Function
PMEPR	Peak-to-Mean Envelope Power Ratio
PN	Pseudo-Noise
SNR	Signal-to-Noise Ratio
STO	Symbol Timing Offset
ZAC	Zero Autocorrelation
ZC	Zadoff–Chu
